# Robotic Gastrectomy and Delivery of Adjuvant Systemic Therapy in Locally Advanced Gastric Adenocarcinoma: An NCDB Propensity Score-Matched Analysis

**DOI:** 10.3390/cancers18071073

**Published:** 2026-03-26

**Authors:** Joseph Broderick, Jun Okui, Paul Mansfield, Hop S. Tran Cao, Brian D. Badgwell, Naruhiko Ikoma

**Affiliations:** Department of Surgical Oncology, The University of Texas MD Anderson Cancer Center, Houston, TX 77030, USA

**Keywords:** gastric cancer, robotic gastrectomy, adjuvant chemotherapy, minimally invasive surgery, National Cancer Database

## Abstract

**Background/Objectives**: Completion of perioperative systemic therapy is essential for improving survival in patients with locally advanced gastric adenocarcinoma; however, many patients do not receive planned adjuvant therapy because of surgical complications or inadequate recovery. Robotic gastrectomy may improve postoperative recovery and facilitate adjuvant therapy delivery, but contemporary national data remain limited. This study evaluated the association between surgical approach and adjuvant systemic therapy utilization. **Methods**: Adults with non-metastatic, locally advanced (>pT2N0 or received neoadjuvant chemotherapy) gastric adenocarcinoma who underwent gastrectomy from 2016 to 2021 were identified from the National Cancer Database. Patients who met the criteria for adjuvant systemic therapy were included. Propensity score matching was performed to compare robotic gastrectomy (RG) with laparoscopic gastrectomy (LG) and open gastrectomy (OG). The primary outcome was receipt of adjuvant systemic therapy (ASTx). The secondary outcomes included days from surgery to ASTx initiation, perioperative outcomes, oncologic quality metrics, and overall survival. **Results**: Among 5853 eligible patients, 17.8% underwent RG. After propensity score matching, ASTx utilization was similar between RG and LG (43.6% vs. 43.9%, *p* = 0.946) and between RG and OG (44.5% vs. 48.0%, *p* = 0.144), with no differences in days from surgery to ASTx initiation. Compared with LG, RG was associated with higher R0 resection rates but higher unplanned 30-day readmission rates. Compared with OG, RG was associated with higher R0 resection rates, greater regional lymph node examination, shorter length of stay, and lower 90-day mortality rates. Overall survival rates did not significantly differ between approaches. **Conclusions**: In this contemporary national analysis, RG did not result in improved delivery or timing of adjuvant systemic therapy despite favorable perioperative outcomes. These findings suggest that considering surgical approach alone is insufficient to address barriers to completion of multimodality therapy in gastric cancer.

## Simple Summary

Surgery for gastric cancer is often combined with chemotherapy to improve survival, but many patients are unable to receive all planned treatments after surgery because of complications or slow recovery. Robotic surgery has been increasingly used for gastric cancer and may help patients recover faster, but it is unclear whether it leads to a higher chemotherapy delivery rate after surgery. In this study, we analyzed a large national database of patients in the United States who underwent gastric cancer surgery and compared robotic, laparoscopic, and open surgical approaches. We found that robotic surgery was associated with modest improvements in short-term recovery, such as shorter hospital stays, but it did not increase the likelihood that patients received chemotherapy after surgery, nor did it improve long-term survival. These findings suggest that while robotic surgery may offer technical and recovery advantages, improving access to and completion of chemotherapy will require broader, team-based approaches beyond the choice of surgical technique alone.

## 1. Introduction

Gastric cancer is the fifth most commonly diagnosed cancer and the fourth leading cause of cancer death worldwide [1]. In the U.S., over 30,000 new cases were estimated to have been diagnosed in 2025, with more than 50% diagnosed at advanced stages [2,3]. The mainstay of treatment for locally advanced gastric cancer is perioperative systemic therapy and gastrectomy with D2 lymphadenectomy, with proven survival benefits from the addition of systemic therapy compared to surgery alone [3,4,5,6,7,8,9,10]. However, gastrectomy can be associated with significant morbidity and prolonged recovery, with only 50–60% of patients ultimately receiving adjuvant systemic therapy [9,10,11]. Furthermore, among those that do receive adjuvant therapy, studies have suggested that delays in starting treatment lead to worse outcomes, although the exact timing and the true effects of adjuvant timing have been debated [12,13,14,15,16].

Minimally invasive approaches to gastrectomy have demonstrated the potential for improved outcomes compared to open gastrectomy (OG), without sacrificing oncologic adequacy. Reported benefits include shorter hospital length of stay (LOS), reduced perioperative morbidity, and lower readmission rates, although results have varied across studies and patient populations [17,18,19,20,21]. These advantages may translate into improved recovery and a greater likelihood of receiving adjuvant therapy, a benefit that has been observed in other malignancies treated with minimally invasive surgical approaches [22,23,24,25]. The use of robotic gastrectomy (RG) for gastric cancer has risen significantly in the past decade, with more than one in four gastrectomies now being performed robotically [26]. Despite this, data evaluating the impact of RG on postoperative recovery and downstream oncologic care—particularly use of adjuvant therapy—remain limited. Accordingly, the aim of this study was to evaluate the association between surgical approach and use of adjuvant therapy among patients with locally advanced gastric adenocarcinoma using data from a contemporary national database.

## 2. Materials and Methods

### 2.1. Study Design and Patients

This study used data from the 2021 National Cancer Database (NCDB) gastric Participant User File. The NCDB is a nationwide, hospital-based oncology database comprising information from over 1500 Commission on Cancer-accredited facilities in the United States. Trained registrars abstract data from patient medical records, with the database capturing approximately 70% of all newly diagnosed cancers in the United States [27,28,29]. The Institutional Review Board at the University of Texas MD Anderson Cancer Center (protocol 2020-0512) approved this study. Tumor staging was defined using the seventh and eighth editions of the American Joint Committee on Cancer staging manual. Gastrectomy was defined by surgery procedure of primary site codes 30–33 (partial gastrectomy), 40–42 (near-total or total gastrectomy), and 50–52 (gastrectomy with removal of a portion of the esophagus). We identified patients diagnosed with gastric adenocarcinoma from 2016 to 2021, defined by the International Classification of Diseases for Oncology third edition codes C16.0–C16.9 with the following tumor histology codes: 8140/3, 8141/3, 8142/3, 8143/3, 8144/3, 8145/3, 8210/3, 8213/3, 8214/3, 8221/3, 8255/3, 8260/3, 8261/3, 8262/3, 8263/3, 8480/3, 8481/3, and 8490/3. We included adult men and women with locally advanced disease without evidence of distant metastasis who underwent gastrectomy and required adjuvant systemic treatment. Patients with locally advanced gastric adenocarcinoma were included if they met any of the following criteria: pT3 or pT4a tumors, any positive pN staging, margin positivity after resection, or if they received neoadjuvant systemic therapy. These criteria were selected to identify patients for whom adjuvant systemic therapy would be indicated under contemporary treatment paradigms [9,11,30]. Patients were excluded if they had metastatic disease, T4b tumors, lacked documented cT or cN stage, lacked a documented surgical approach, if they received neoadjuvant immunotherapy or radiation therapy, or if they underwent multi-organ resection at the time of surgery (other than a portion of the esophagus).

### 2.2. Outcomes

Patients were grouped and compared on the basis of the surgical approach: OG vs. RG; laparoscopic gastrectomy (LG) vs. RG. The primary outcome was the use of adjuvant systemic therapy (ASTx) based on a treatment sequence code [RX_SUMM_SYSTEMIC_SUR_SEQ]. Secondary outcomes include days from surgery to ASTx, the proportion of patients who started systemic therapy ≤8 weeks after surgery (ASTx < 8w) among those who received ASTx, LOS, 30-day readmission, R0 resection rates, regional lymph nodes examined (RLNE), 30- and 90-day mortality rates, and 3-year overall survival rates.

### 2.3. Statistical Analysis

To balance the clinicopathological characteristics between the surgical approach (OG vs. RG; LG vs. RG),we used propensity score (PS) matching to compare outcomes. Because the present study was not designed to evaluate three-group comparisons, but rather to specifically assess differences between the robotic and non-robotic approaches, only pairwise comparisons were performed. The PS was estimated using logistic regression models predicting whether patients would receive LG or OG vs. RG on the basis of baseline variables (age; sex; race; year of diagnosis; Charlson Comorbidity Index Score; insurance type; facility type; clinical T and N categories; tumor location; neoadjuvant chemotherapy [yes or no]; histological type; gastrectomy type). After PS estimation, each patient undergoing LG or OG was matched to a patient undergoing RG using the one-to-one nearest-neighbor matching algorithm, without replacement and with a matching caliper of 0.2 SD of the PS. We calculated the standardized mean difference to assess the balance of the two matched groups. Standardized mean differences above 0.1 were considered to indicate an imbalance [31,32].

Continuous variables were presented as means and SDs and analyzed using the Welch’s *t*-test, while categorical variables were presented as frequencies and proportions and analyzed using the chi-square test. R software v.4.5.1 (The R Foundation for Statistical Computing, Vienna, Austria) was used to perform all statistical analyses. Missing values were not imputed. All *p*-values were two-tailed, with statistical significance set at *p* < 0.05.

## 3. Results

### 3.1. Total Cohort

**Figure 1:**
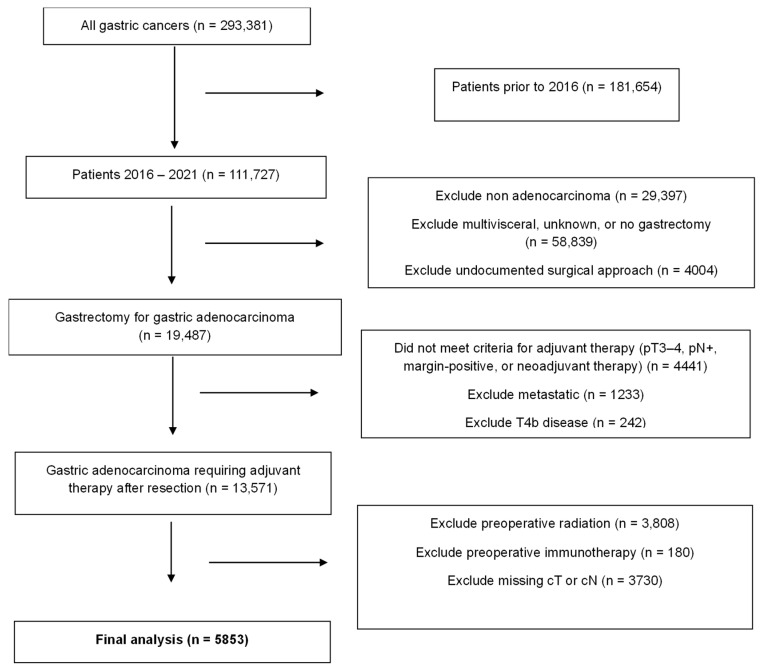
Patient selection flow chart.

Overall, 5853 patients met our inclusion criteria. The patient selection flow chart can be found in Figure 1. The cohort was predominately non-Hispanic White (51%) and male (63.4%) with a mean age of about 65 years. Most were from a metropolitan area (89.5%) with government insurance (62.8%) treated at an academic facility (54.4%). The majority had a Charlson Comorbidity Index Score of 0 (67.2%). Patients most commonly had clinical stage 2 disease (42.6%), T3 (50.9%), or N0 (61.4%). Tumor location was fairly evenly distributed throughout the stomach, with slightly more found in the fundus/body (31.1%). Most patients underwent partial gastrectomy (56.9%), with the majority (53.0%) undergoing OG, followed by LG (29.2%) and RG (17.8%). Most (65.3%) patients received neoadjuvant chemotherapy. Patient characteristics, as well as tumor and treatment characteristics, can be found in Table 1 and Table 2, respectively.

### 3.2. Propensity Score-Matched Cohorts

#### 3.2.1. Patient, Tumor and Treatment Characteristics

The patient, tumor and treatment characteristics of the PS-matched cohorts can be found in Table 3 and Table 4. No significant differences in matched patient, tumor or treatment characteristics were observed by surgical approach (RG vs. LG, RG vs. OG), although the LG and OG cohorts had significantly larger tumor sizes than their matched RG cohorts (LG vs. RG—49.84 vs. 35.00 cm, *p* = 0.002; OG vs. RG—43.32 vs. 34.85 cm, *p* = 0.006). Accordingly, there was a statistically significant difference in pathological T staging between OG and RG, with the OG cohort having a higher percentage of T3 and T4 tumors (40.2% and 26.1% vs. 38.0% and 19.2%, respectively; *p* = 0.034). There was otherwise no significant difference in pathological staging variables, including nodal status, nodal positivity, and pathological stage, between the RG vs. LG and the RG vs. OG cohort.

#### 3.2.2. RG vs. LG Outcomes

**Figure 2:**
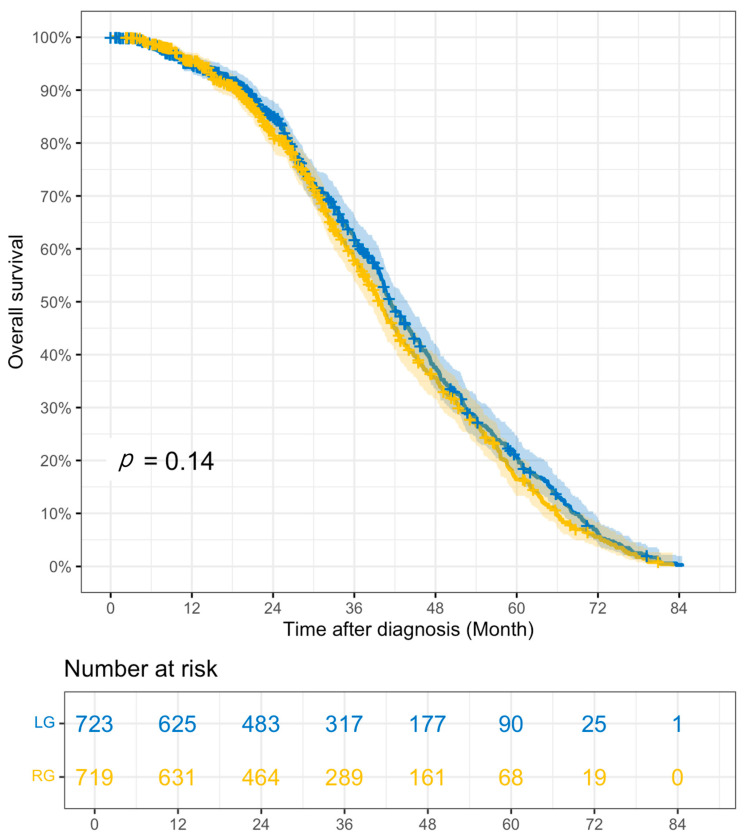
Kaplan–Meier survival curve RG vs. LG PSM cohorts. RG—robotic gastrectomy; LG—laparoscopic gastrectomy.

The outcomes of the PS-matched cohort comparisons in RG vs. LG can be found in Table 5. When comparing RG vs. LG, ASTx use did not significantly differ between groups (43.6% vs. 43.9%, *p* = 0.946), nor did the days from surgery to ASTx (57.13 vs. 60.44 days, *p* = 0.353) or the proportion with ASTx < 8w (60.0% vs. 60.8%, *p* = 0.998). RG was associated with significantly higher rates of R0 resection than LG (93.6% vs. 88.9%, *p* < 0.001), while the number of RLNE was similar between groups (24.68 vs. 24.77, *p* = 0.900). RG was also associated with higher rates of unplanned 30-day hospital readmission (6.7% vs. 4.4%, *p* = 0.041), whereas LOS was comparable (8.42 vs. 8.37 days, *p* = 0.919). There were no significant differences in 30- or 90-day mortality rates between approaches, and overall survival rates were similar (3-year overall survival: 57.8% [53.8–62.1%] vs. 62.1% [58.2–66.3%], *p* = 0.14), including when stratified by stage. The Kaplan–Meier survival curve for RG vs. LG can be found in Figure 2.

#### 3.2.3. RG vs. OG Outcomes

**Figure 3:**
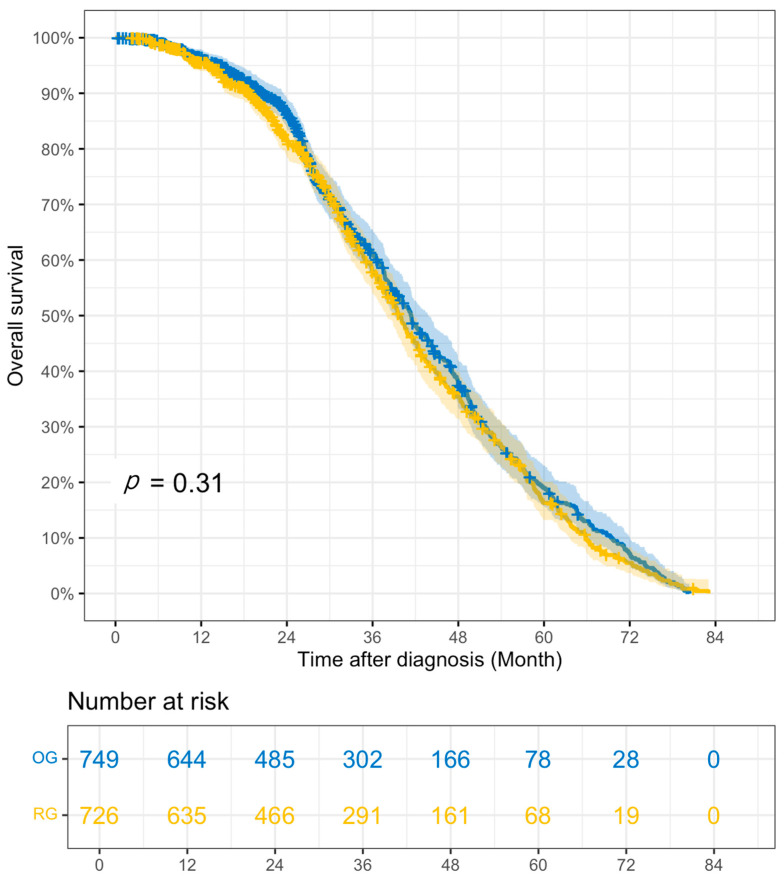
Kaplan–Meier survival curve of RG vs. OG PSM cohorts. RG—robotic gastrectomy; LG—laparoscopic gastrectomy.

The outcomes of the PS-matched cohort comparisons in RG vs. OG are shown in Table 5. ASTx use was similar between groups (44.5% vs. 48.0%, *p* = 0.144), as were the days from surgery to ASTx (56.59 vs. 58.35 days, *p* = 0.558) and the proportion of patients with ASTx < 8w (60.9% vs. 55.4%, *p* = 0.425). RG was associated with higher rates of R0 resection (93.3% vs. 89.4%, *p* = 0.004), a greater number of RLNE (24.68 vs. 22.91, *p* = 0.004), and a significantly shorter LOS than OG (8.28 vs. 9.20 days, *p* = 0.019). Unplanned 30-day readmission rates did not differ between groups (6.9% vs. 5.6%, *p* = 0.294). While 30-day mortality rates were similar (1.5% vs. 2.0%, *p* = 0.615), RG was associated with a lower 90-day mortality rate than OG (3.2% vs. 5.8%, *p* = 0.025). There were no significant differences in overall survival rates between the two approaches (3-year overall survival: 57.9% [53.9–62.1] vs. 61.3% [57.4–65.5], *p* = 0.31), including when stratified by stage. The Kaplan–Meier survival curve for RG vs. OG can be found in Figure 3.

## 4. Discussion

In this contemporary NCDB analysis of patients with locally advanced gastric adenocarcinoma, we found that RG was not associated with higher rates of ASTx use or earlier initiation of ASTx than LG or OG. Despite modest improvements in selected perioperative and oncologic quality metrics—including R0 resection rates, RLNE, LOS, and 90-day mortality rates—RG did not translate into measurable downstream gains in the receipt or timing of ASTx, nor into differences in overall survival rates. These findings underscore the complexity of postoperative recovery and oncologic care delivery in gastric cancer and suggest that surgical approach alone is insufficient to overcome the barriers to completion of multimodality therapy.

Prior NCDB analyses and institutional series have identified patient comorbidities and postoperative morbidity as key drivers of adjuvant therapy omission or delay [29,33,34,35]. Minimally invasive gastrectomy has been proposed as a strategy to mitigate these barriers, with institutional studies reporting lower postoperative complication rates and improved return to intended oncologic therapy compared with open surgery [8,17,36]. However, large national database analyses have not consistently demonstrated an association between minimally invasive approaches and increased ASTx utilization [18,34].

Among minimally invasive approaches, the impact of RG on ASTx delivery has not been well studied, despite its rapid adoption in recent years. Available evidence suggests that while RG may facilitate slightly earlier initiation of adjuvant chemotherapy than LG, this does not translate into higher overall utilization of adjuvant systemic therapy [37,38]. Specifically, one randomized controlled trial and one retrospective study from China demonstrated modest reductions in time to adjuvant chemotherapy initiation following robotic distal gastrectomy—on the order of 2–4 days—differences that are unlikely to be clinically meaningful, while showing no significant differences in receipt of overall adjuvant therapy [37,38]. Similarly, a U.S.-based NCDB analysis reported no difference in postoperative chemotherapy utilization between RG and LG, although this study included all stages of gastric adenocarcinoma and spanned 2010–2019, a period marked by evolving surgical approaches and systemic therapy paradigms [39].

In this context, the present study evaluates RG within a contemporary national cohort during its rapid adoption from 2016 to 2021, a period during which utilization of the robotic approach nearly quadrupled compared with the preceding five years [39]. Despite evidence that RG is associated with improvements in select perioperative outcomes in this and prior studies [19,20,40], these advantages did not translate into increased ASTx use. Collectively, these findings suggest that while RG may improve aspects of perioperative care and recovery, it does not meaningfully alter the downstream delivery of systemic therapy in contemporary practice.

The modern paradigm for perioperative systemic therapy in locally advanced gastric cancer has evolved from the MAGIC trial, which demonstrated improved survival with perioperative chemotherapy, to contemporary regimens such as FLOT, which have further improved outcomes and now represent a standard of care [9,11]. More recently, emerging data incorporating immunotherapy into perioperative treatment strategies continue to refine this approach [41]. Importantly, survival outcomes are closely tied not only to the receipt of systemic therapy but also the type, completion, and intensity. While the NCDB captures whether adjuvant systemic therapy was initiated, it does not provide information on specific regimens or the number of cycles administered, and therefore, our analysis reflects treatment initiation rather than treatment completeness or intensity. Furthermore, successful delivery of adjuvant therapy is inherently multifactorial. Patients with gastric cancer often present with baseline malnutrition and physiologic deconditioning, requiring multidisciplinary evaluation and optimization prior to treatment. Postoperatively, recovery is strongly influenced by nutritional support, adherence to enhanced recovery after surgery (ERAS) pathways, and coordination of care [42]. The multifactorial nature of successful adjuvant therapy delivery, much of which is not fully captured in administrative datasets despite PS matching—including nutritional and performance status, patient frailty, social support, specific comorbidities, postoperative complications, and broader access to care factors—has been demonstrated across a variety of malignancies [43,44,45,46,47,48,49]. These considerations likely explain why improvements in perioperative outcomes associated with minimally invasive or robotic approaches do not necessarily translate into higher rates of adjuvant therapy utilization.

Although RG was not associated with improved ASTx delivery, it was associated with higher R0 resection rates and, compared with OG, more RLNE. Prior studies have reported higher lymph node yield with RG relative to both OG and LG [37,38,40,50,51,52,53]; however, in the present analysis, RLNE did not differ between RG and LG, suggesting that the observed differences may reflect minimally invasive versus open approaches rather than a robotic approach. Contemporary factors—including patient selection, institutional volume, pathologic processing, and increasing standardization of D2 lymphadenectomy—may further attenuate measurable differences between RG and LG in national cohorts. Improvements in R0 resection with RG have been more consistently reported relative to LG than to OG [54,55], and evidence supporting the superiority of RG over OG remains limited to retrospective analyses [20]. Given the similar oncologic exposure afforded by open and robotic approaches, particularly for circumferential margin assessment, the observed association between RG and improved R0 resection compared with OG may reflect residual confounding or practice-pattern differences rather than a true technical advantage and should be interpreted cautiously.

RG was associated with a shorter LOS and lower 90-day mortality rate than OG, consistent with prior reports demonstrating a shorter LOS with minimally invasive gastrectomy [17,18,20,34,50]. In contrast, evidence supporting lower postoperative mortality rates with RG remains limited to retrospective registry-based studies [20]. Notably, RG was associated with higher 30-day readmission rates than LG, a finding that contrasts with much of the existing literature [19,38,40,51,52,54,55,56]. This discrepancy may reflect differences in referral center case mix, hospital volume, and postoperative care pathways rather than inferior perioperative quality. In particular, high-volume centers may employ closer postoperative surveillance and maintain a lower threshold for readmission to manage early complications—especially in nutritionally vulnerable gastric cancer patients—which may increase readmission rates without adversely affecting overall outcomes. There is, however, evidence to suggest that increased adoption of the robotic platform has resulted in its increased utilization in select operations [57,58]. This could have important downstream effects on postoperative outcomes if adoptions occur in the absence of experience, with RG shown to have a shorter learning curve than LG, though this has not yet been shown in the gastrectomy literature [59]. Importantly, no improvement in long-term survival was observed with RG. Taken together, these findings suggest that while RG may confer short-term perioperative advantages over OG, postoperative care trajectories following minimally invasive gastrectomy remain heterogeneous at the national level, and registry-based readmission metrics may be influenced by health system factors rather than by true differences in complication burden.

This study has several important limitations. First, the retrospective nature of NCDB analyses introduces the potential for residual confounding despite PS matching. Second, the NCDB lacks granular data on postoperative complications, nutritional status, performance status, and specific chemotherapy regimens, all of which strongly influence adjuvant therapy delivery. Third, treatment sequence codes may have misclassified some patients, particularly those who experienced early recurrence or treatment intolerance. Finally, surgeon experience, institutional volume, and ERAS pathway adoption—factors known to influence outcomes—are not fully captured in the database.

## 5. Conclusions

RG may offer perioperative and technical advantages compared with LG and OG, but it was not associated with improved delivery of ASTx in this study of U.S. patients with locally advanced gastric cancer. These findings suggest that optimizing the surgical approach alone is insufficient to overcome persistent barriers to multimodality therapy completion. Future studies should evaluate integrated strategies combining robotic surgery with standardized enhanced recovery protocols and multidisciplinary patient care to meaningfully improve completion of intended oncologic therapy and long-term outcomes.

## Figures and Tables

**Table 1:** Patient characteristics.

	Total	Laparoscopic	Open	Robotic	*p*
Surgical approach, no. (%)	5853	1709 (29.2)	3104 (53.0)	1040 (17.8)	
Age (mean (SD))	64.96 (12.21)	65.33 (12.24)	64.98 (12.12)	64.31 (12.41)	0.103
Sex—male, no. (%)	3710 (63.4)	1094 (64.0)	1977 (63.7)	639 (61.4)	0.348
Race, no. (%)					<0.001
Hispanic	908 (15.6)	271 (15.9)	465 (15.1)	172 (16.7)	
Non-Hispanic Asian	793 (13.6)	286 (16.8)	335 (10.9)	172 (16.7)	
Non-Hispanic Black	1011 (17.4)	247 (14.5)	622 (20.2)	142 (13.8)	
Non-Hispanic White	2970 (51.1)	858 (50.4)	1586 (51.5)	526 (51.0)	
Other	132 (2.3)	41 (2.4)	71 (2.3)	20 (1.9)	
Year of diagnosis, no. (%)					<0.001
2016	1012 (17.3)	324 (19.0)	589 (19.0)	99 (9.5)	
2017	1010 (17.3)	304 (17.8)	577 (18.6)	129 (12.4)	
2018	1009 (17.2)	263 (15.4)	562 (18.1)	184 (17.7)	
2019	1038 (17.7)	296 (17.3)	544 (17.5)	198 (19.0)	
2020	855 (14.6)	256 (15.0)	437 (14.1)	162 (15.6)	
2021	929 (15.9)	266 (15.6)	395 (12.7)	268 (25.8)	
Charlson Comorbidity Index Score, no. (%)					0.177
0	3935 (67.2)	1167 (68.3)	2069 (66.7)	699 (67.2)	
1	1163 (19.9)	326 (9.1)	618 (19.9)	219 (21.1)	
2	390 (6.7)	100 (5.9)	231 (7.4)	59 (5.7)	
3	365 (6.2)	116 (6.8)	186 (6.0)	63 (6.1)	
Insurance status, no. (%)					0.003
Government	3636 (62.8)	1088 (64.4)	1915 (62.5)	633 (61.3)	
No insurance	174 (3.0)	30 (1.8)	114 (3.7)	30 (2.9)	
Private	1977 (34.2)	571 (33.8)	1037 (33.8)	369 (35.8)	
Treatment facility type, no. (%)					<0.001
Academic program	3067 (54.4)	930 (56.3)	1594 (53.2)	543 (54.6)	
Community cancer program	1620 (28.7)	472 (28.6)	910 (30.4)	238 (23.9)	
Integrated network cancer program	956 (16.9)	250 (15.1)	492 (16.4)	214 (21.5)	
Median income quartile, no. (%) *					0.002
1st quartile	941 (19.4)	253 (17.8)	539 (20.9)	149 (17.1)	
2nd quartile	959 (19.7)	250 (17.6)	535 (20.8)	174 (20.0)	
3rd quartile	1079 (22.2)	313 (22.1)	561 (21.8)	205 (23.6)	
4th quartile	1884 (38.7)	602 (42.5)	941 (36.5)	341 (39.2)	
Educational attainment, no. (%) **					0.263
1st quartile	888 (18.2)	278 (19.6)	454 (17.6)	156 (17.9)	
2nd quartile	1283 (26.3)	357 (25.1)	715 (27.7)	211 (24.3)	
3rd quartile	1303 (26.7)	371 (26.1)	684 (26.5)	248 (28.5)	
4th quartile	1400 (28.7)	414 (29.2)	731 (28.3)	255 (29.3)	
Urban/rural, no. (%)					0.001
Metro	5034 (89.5)	1502 (91.1)	2620 (88.0)	912 (91.7)	
Rural	70 (1.2)	16 (1.0)	48 (1.6)	6 (0.6)	
Urban	518 (9.2)	131 (7.9)	310 (10.4)	77 (7.7)	

* Measure of median income for each patient’s area of residence, categorized into quartiles among all U.S. zip codes based on data from 2016 to 2020. ** Measure of educational attainment for each patient’s area of residence, categorized into quartiles among all U.S. zip codes based on data from 2016 to 2020.

**Table 2:** Tumor and treatment characteristics.

	Total	Laparoscopic	Open	Robotic	*p*
Tumor size, mean (SD)	47.02 (37.76)	46.13 (32.62)	52.14 (44.31)	34.49 (17.11)	<0.001
Clinical T staging, no. (%)					0.232
T1	869 (14.8)	259 (15.2)	460 (14.8)	150 (14.4)	
T2	1609 (27.5)	484 (28.3)	818 (26.4)	307 (29.5)	
T3	2977 (50.9)	861 (50.4)	1595 (51.4)	521 (50.1)	
T4	398 (6.8)	105 (6.1)	231 (7.4)	62 (6.0)	
Clinical N staging, no. (%)					0.017
N0	3591 (61.4)	1059 (62.0)	1900 (61.2)	632 (60.8)	
N1	1614 (27.6)	464 (27.2)	834 (26.9)	316 (30.4)	
N2	545 (9.3)	156 (9.1)	304 (9.8)	85 (8.2)	
N3	103 (1.8)	30 (1.8)	66 (2.1)	7 (0.7)	
Clinical stage, no. (%)					0.065
1	1747 (30.2)	534 (31.7)	890 (29.0)	323 (31.4)	
2	2463 (42.6)	697 (41.4)	1356 (44.3)	410 (39.8)	
3	1569 (27.2)	454 (26.9)	818 (26.7)	297 (28.8)	
Location of primary tumor, no. (%)					0.001
Antrum/pylorus	1661 (28.4)	488 (28.6)	889 (28.6)	284 (27.3)	
Cardia	1362 (23.3)	439 (25.7)	674 (21.7)	249 (23.9)	
Fundus/body	1823 (31.1)	515 (30.1)	950 (30.6)	358 (34.4)	
Overlap	531 (9.1)	139 (8.1)	311 (10.0)	81 (7.8)	
Stomach NOS	476 (8.1)	128 (7.5)	280 (9.0)	68 (6.5)	
Adenocarcinoma type, no. (%)					0.764
Adenocarcinoma, NOS	3161 (54.0)	914 (53.5)	1668 (53.7)	579 (55.7)	
Diffuse	1537 (26.3)	454 (26.6)	819 (26.4)	264 (25.4)	
Intestinal	874 (14.9)	261 (15.3)	464 (14.9)	149 (14.3)	
Mixed	192 (3.3)	57 (3.3)	98 (3.2)	37 (3.6)	
Mucinous	89 (1.5)	23 (1.3)	55 (1.8)	11 (1.1)	
Gastrectomy type, no. (%)					<0.001
Partial gastrectomy	3333 (56.9)	1002 (58.6)	1737 (56.0)	594 (57.1)	
Total or near total	1287 (22.0)	316 (18.5)	752 (24.2)	219 (21.1)	
Gastrectomy with a portion of esophagus	1233 (21.1)	391 (22.9)	615 (19.8)	227 (21.8)	
Neoadjuvant chemo, no. (%)	3824 (65.3)	1093 (64.0)	1996 (64.3)	735 (70.7)	<0.001

NOS—not otherwise specified.

**Table 3:** Characteristics of patients in propensity score-matched cohorts.

	Robotic	Laparoscopic	*p*	Robotic	Open	*p*
n	933	933		961	961	
Age (mean (SD))	65.95 (10.83)	65.98 (11.24)	0.955	65.79 (10.91)	65.54 (11.10)	0.622
Sex—male, no. (%)	584 (62.6)	569 (61.0)	0.505	596 (62.0)	605 (63.0)	0.706
Race, no. (%)			0.797			0.847
Hispanic	145 (15.5)	159 (17.0)		156 (16.2)	166 (17.3)	
Non-Hispanic Asian	158 (16.9)	148 (15.9)		150 (15.6)	160 (16.6)	
Non-Hispanic Black	132 (14.1)	140 (15.0)		134 (13.9)	121 (12.6)	
Non-Hispanic White	479 (51.3)	464 (49.7)		502 (52.2)	494 (51.4)	
Other	19 (2.0)	22 (2.4)		19 (2.0)	20 (2.1)	
Year of diagnosis, no. (%)			0.998			0.881
2016	93 (10.0)	91 (9.8)		93 (9.7)	100 (10.4)	
2017	125 (13.4)	132 (14.1)		125 (13.0)	133 (13.8)	
2018	170 (18.2)	170 (18.2)		171 (17.8)	175 (18.2)	
2019	179 (19.2)	181 (19.4)		183 (19.0)	184 (19.1)	
2020	152 (16.3)	149 (16.0)		154 (16.0)	157 (16.3)	
2021	214 (22.9)	210 (22.5)		235 (24.5)	212 (22.1)	
Charlson Comorbidity Index Score, no. (%)			0.992			0.83
0	621 (66.6)	623 (66.8)		637 (66.3)	635 (66.1)	
1	195 (20.9)	195 (20.9)		203 (21.1)	198 (20.6)	
2	55 (5.9)	52 (5.6)		58 (6.0)	55 (5.7)	
3	62 (6.6)	63 (6.8)		63 (6.6)	73 (7.6)	
Insurance status, no. (%)			0.841			0.971
Government	593 (63.6)	604 (64.7)		606 (63.1)	601 (62.5)	
No insurance	18 (1.9)	16 (1.7)		24 (2.5)	24 (2.5)	
Private	322 (34.5)	313 (33.5)		331 (34.4)	336 (35.0)	
Treatment facility type, no. (%)			0.782			0.851
Academic or research program	520 (55.7)	510 (54.7)		527 (54.8)	522 (54.3)	
Community cancer program	233 (25.0)	231 (24.8)		234 (24.3)	229 (23.8)	
Integrated network cancer program	180 (19.3)	192 (20.6)		200 (20.8)	210 (21.9)	
Median income quartile, no. (%) *			0.105			0.196
1st quartile	135 (17.3)	150 (19.8)		143 (17.7)	168 (21.0)	
2nd quartile	155 (19.8)	119 (15.7)		160 (19.8)	172 (21.5)	
3rd quartile	188 (24.1)	173 (22.8)		189 (23.4)	165 (20.6)	
4th quartile	303 (38.8)	317 (41.8)		316 (39.1)	296 (37.0)	
Educational attainment, no. (%) **			0.2			0.22
1st quartile	138 (17.6)	151 (19.9)		144 (17.8)	141 (17.6)	
2nd quartile	185 (23.7)	195 (25.7)		192 (23.7)	220 (27.4)	
3rd quartile	227 (29.0)	186 (24.5)		235 (29.0)	202 (25.2)	
4th quartile	232 (29.7)	228 (30.0)		238 (29.4)	240 (29.9)	
Urban/rural, no. (%)			0.863			0.026
Metro	812 (91.1)	822 (91.6)		839 (91.3)	803 (87.8)	
Rural	6 (0.7)	7 (0.8)		6 (0.7)	14 (1.5)	
Urban	73 (8.2)	68 (7.6)		74 (8.1)	98 (10.7)	

* Measure of median income for each patient’s area of residence, categorized into quartiles among all U.S. zip codes based on data from 2016 to 2020. ** Measure of educational attainment for each patient’s area of residence, categorized into quartiles among all U.S. zip codes based on data from 2016 to 2020.

**Table 4:** Tumor and treatment characteristics in propensity score-matched cohorts.

	Robotic	Laparoscopic	*p*	Robotic	Open	*p*
Tumor size, mean (SD)	35.00 (17.49)	49.84 (38.29)	0.002	34.85 (17.44)	43.32 (21.33)	0.006
Clinical T staging, no. (%)			0.981			0.911
T1	134 (14.4)	133 (14.3)		139 (14.5)	148 (15.4)	
T2	270 (28.9)	272 (29.2)		276 (28.7)	277 (28.8)	
T3	473 (50.7)	468 (50.2)		487 (50.7)	474 (49.3)	
T4	56 (6.0)	60 (6.4)		59 (6.1)	62 (6.5)	
Clinical N staging, no. (%)			0.988			0.979
N0	574 (61.5)	571 (61.2)		592 (61.6)	584 (60.8)	
N1	278 (29.8)	282 (30.2)		284 (29.6)	289 (30.1)	
N2	74 (7.9)	72 (7.7)		78 (8.1)	80 (8.3)	
N3	7 (0.8)	8 (0.9)		7 (0.7)	8 (0.8)	
Clinical stage, no. (%)			0.716			0.381
1	289 (31.3)	284 (30.9)		299 (31.4)	277 (29.2)	
2	367 (39.8)	381 (41.5)		379 (39.9)	407 (42.8)	
3	267 (28.9)	253 (27.6)		273 (28.7)	266 (28.0)	
Location of primary tumor, no. (%)			0.911			0.952
Antrum/pylorus	259 (27.8)	259 (27.8)		267 (27.8)	253 (26.3)	
Cardia	228 (24.4)	217 (23.3)		229 (23.8)	240 (25.0)	
Fundus/body	304 (32.6)	304 (32.6)		322 (33.5)	326 (33.9)	
Overlap	77 (8.3)	78 (8.4)		78 (8.1)	76 (7.9)	
Stomach NOS	65 (7.0)	75 (8.0)		65 (6.8)	66 (6.9)	
Adenocarcinoma type, no. (%)			0.968			0.848
Adenocarcinoma, NOS	524 (56.2)	515 (55.2)		537 (55.9)	544 (56.6)	
Diffuse	232 (24.9)	238 (25.5)		239 (24.9)	239 (24.9)	
Intestinal	136 (14.6)	134 (14.4)		140 (14.6)	127 (13.2)	
Mixed	30 (3.2)	35 (3.8)		34 (3.5)	41 (4.3)	
Mucinous	11 (1.2)	11 (1.2)		11 (1.1)	10 (1.0)	
Gastrectomy type, no. (%)			0.671			0.441
Partial gastrectomy	533 (57.1)	549 (58.8)		548 (57.0)	524 (54.5)	
Total or near total	191 (20.5)	190 (20.4)		202 (21.0)	204 (21.2)	
Gastrectomy with a portion of esophagus	209 (22.4)	194 (20.8)		211 (22.0)	233 (24.2)	
Neoadjuvant chemo, no. (%)	647 (69.3)	648 (69.5)	1	670 (69.7)	680 (70.8)	0.653

NOS—not otherwise specified.

**Table 5:** Outcomes of propensity score-matched cohorts.

	Robotic	Laparoscopic	*p*	Robotic	Open	*p*
Pathological T staging, no. (%)			0.405			0.034
T0	19 (4.6)	25 (5.9)		19 (4.5)	18 (4.0)	
T1	88 (21.3)	95 (22.6)		91 (21.6)	68 (15.2)	
T2	69 (16.7)	52 (12.4)		70 (16.6)	65 (14.5)	
T3	158 (38.3)	160 (38.0)		160 (38.0)	180 (40.2)	
T4	79 (19.1)	89 (21.1)		81 (19.2)	117 (26.1)	
Pathological N staging, no. (%)			0.932			0.222
N0	157 (37.9)	164 (39.3)		159 (37.7)	161 (36.0)	
N1	114 (27.5)	108 (25.9)		119 (28.2)	105 (23.5)	
N2	72 (17.4)	76 (18.2)		73 (17.3)	91 (20.4)	
N3	71 (17.1)	69 (16.5)		71 (16.8)	90 (20.1)	
Pathological N stage positivity (N+/N0, no. (%))	257/157 (62.1/37.9)	253/164 (60.7/39.3)	0.73	263/159 (62.3/37.7)	286/161 (64.0/36.0)	0.662
Pathological stage, no. (%)			0.582			0.095
1	64 (16.3)	74 (18.8)		66 (16.5)	61 (14.3)	
2	181 (46.1)	170 (43.1)		185 (46.1)	174 (40.8)	
3	148 (37.7)	150 (38.1)		150 (37.4)	191 (44.8)	
ASTx use, no. (%)	406 (43.6)	408 (43.9)	0.946	427 (44.5)	458 (48.0)	0.144
Days from surgery to ASTx, mean (SD)	57.13 (23.43)	60.44 (32.74)	0.353	56.59 (23.45)	58.35 (26.10)	0.558
ASTx < 8 weeks from surgery, no. (%)	78 (60.0)	76 (60.8)	0.998	81 (60.9)	77 (55.4)	0.425
LOS, mean (SD)	8.42 (8.91)	8.37 (7.79)	0.919	8.28 (8.73)	9.20 (8.03)	0.019
Unplanned 30 d hospital readmission, no. (%)	62 (6.7)	41 (4.4)	0.041	66 (6.9)	54 (5.6)	0.294
Negative resection margin (R0/R+, no. (%))	864/59 (93.6/6.4)	827/103 (88.9/11.1)	<0.001	887/64 (93.3/6.7)	854/101 (89.4/10.6)	0.004
RLNE, no. (%)	24.68 (13.78)	24.77 (14.39)	0.9	24.68 (13.74)	22.91 (12.66)	0.004
30 d mortality, no. (%)	11 (1.5)	15 (2.1)	0.565	11 (1.5)	15 (2.0)	0.615
90 d mortality, no. (%)	23 (3.2)	38 (5.4)	0.068	23 (3.2)	43 (5.8)	0.025
3 yr overall survival, % (95% CI) *	57.8% (53.8–62.1)	62.1% (58.2–66.3)	0.14	57.9% (53.9–62.1)	61.3% (57.4–65.5)	0.31
Clinical Stage 1	56.1% (95% CI, 49.3–63.8%)	60.1% (95% CI, 53.2–68.0%)	0.91	56.0% (95% CI, 49.3–63.7%)	58.0% (95% CI, 51.0–65.9%)	0.67
Clinical Stage 2	60.7% (95% CI, 54.4–67.6%)	62.2% (95% CI, 56.4–68.6%)	0.30	60.9% (95% CI, 54.7–67.8%)	65.9% (95% CI, 60.2–72.2%)	0.35
Clinical Stage 3	56.4% (95% CI, 49.0–64.9%)	64.1% (95% CI, 56.5–72.7%)	0.28	56.4% (95% CI, 49.0–64.9%)	57.4% (95% CI, 49.6–66.4%)	0.25

ASTx—adjuvant systemic therapy; LOS—length of stay after surgical procedure; RLNE—regional lymph nodes examined. * *p*-values for survival derived from log-rank Kaplan–Meier curves. Dividing PSM cohorts into clinical stages for subgroup survival analysis eliminates the balance and introduces confounding.

## Data Availability

The data is available in the 2021 National Cancer Database (NCDB) Participant User Data File (PUF). Application for access can be submitted at https://www.facs.org/quality-programs/cancer-programs/national-cancer-database/puf/ (accessed on 1 August 2025).

## Abbreviations

The following abbreviations are used in this manuscript:

NCDB	National Cancer Database
NOS	Not otherwise specified
ASTx	Adjuvant systemic therapy
ASTx < 8w	Adjuvant systemic therapy started <8 weeks after surgery
RLNE	Regional lymph nodes examined
